# Polarized Endocytosis of the Keratinocyte Growth Factor Receptor in Migrating Cells: Role of Src-Signaling and Cortactin

**DOI:** 10.1371/journal.pone.0029159

**Published:** 2011-12-14

**Authors:** Francesca Belleudi, Cristina Scrofani, Maria Rosaria Torrisi, Patrizia Mancini

**Affiliations:** 1 Istituto Pasteur-Fondazione Cenci Bolognetti, Dipartimento di Medicina Clinica e Molecolare, Sapienza Università di Roma, Roma, Italy; 2 Azienda Ospedaliera S. Andrea, Roma, Italy; 3 Dipartimento di Medicina Sperimentale, Sapienza Università di Roma, Roma, Italy; University of Birmingham, United Kingdom

## Abstract

Cell migration is a physiological process that requires endocytic trafficking and polarization of adhesion molecules and receptor tyrosine kinases (RTKs) to the leading edge. Many growth factors are able to induce motility by binding to specific RTK on target cells. Among them, keratinocyte growth factor (KGF or FGF7) and fibroblast growth factor 10 (FGF10), members of the FGF family, are motogenic for keratinocytes, and exert their action by binding to the keratinocyte growth factor receptor (KGFR), a splicing variant of FGFR2, exclusively expressed on epithelial cells. Here we analyzed the possible role of cortactin, an F-actin binding protein which is tyrosine phosphorylated by Src and is involved in KGFR-mediated cell migration, in the KGFR endocytosis and polarization to the leading edge of migrating cells upon ligand-induced stimulation. Biochemical phosphorylation study revealed that both KGF and FGF10 were able to induce tyrosine phosphorylation of Src and in turn of cortactin, as demonstrated by using the specific pharmacological Src-inhibitor SU6656, although FGF10 effect was delayed with respect to that promoted by KGF. Immunofluorescence analysis demonstrated the polarized localization of KGFR upon ligand stimulation to the leading edge of migrating keratinocytes, process that was regulated by Src. Moreover, we showed that the colocalization of cortactin with KGFR at the plasma membrane protrusions and on early endosomes after KGF and FGF10 treatment was Src-dependent. Further, by using a RNA interference approach through microinjection, we showed that cortactin is required for KGFR endocytosis and that the clathrin-dependent internalization of the receptor is a critical event for its polarization. Finally, KGFR expression and polarization enhanced cell migration in a scratch assay. Our results indicate that both Src and cortactin play a key role in the KGFR endocytosis and polarization at the leading edge of migrating keratinocytes, supporting the crucial involvement of RTK trafficking in cell motility.

## Introduction

Cell migration is a physiological process that involves actin cytoskeleton remodeling in lamellipodia and membrane ruffles at the leading edge of the cell, and the assembly and disassembly of adhesion contacts at the rear part of the cell [1]. Many studies have described the migratory effect of different growth factors, that exert their activity by binding to specific receptor tyrosine kinases (RTKs) expressed on target cells. During the ligand-stimulated cell motility not only the extracellular matrix receptors, such as integrins [2], but also the activated RTKs [3], [4] are maintained in a polarized state by continuous internalization and recycling events retargeting the receptors to the cell's advancing edge [5]. Although a direct correlation between RTK endocytosis and cell motility has not yet been clarified, studies conducted by different groups suggest a key role of different actin-binding proteins in the regulation of RTKs internalization and consequent polarization following the ligand-dependent motogenic stimulus. One of the proteins that provide a direct link between the actin assembly and the membrane dynamic during receptor-mediated endocytosis is cortactin, an F-acting binding protein initially identified as a major substrate for the protein tyrosine kinase Src [6], [7]. The observations that cortactin is present in lamellipodia and membrane ruffles [8], as well as on endosomal vesicles [9]–[11], suggest its involvement in cytoskeleton organization during the membrane trafficking associated with cell migration. In fact, the role of cortactin in linking transmembrane signaling and cell motility is well recognized [8]. In addition, the role of cortactin in clathrin-mediated endocytosis has been demonstrated by microinjection of anti-cortactin antibodies [12] and by depletion of cortactin using RNA interference [13], leading to inhibition of the internalization of either transferrin or low density lipoproteins (LDL). Moreover, transfection with the dominant-negative mutant of cortactin in combination with cortactin siRNA showed that internalization not only of transferrin, but also of the γc cytokine receptor, was inhibited, suggesting that cortactin is involved in clathrin–independent mechanisms of uptake [14]. Receptor-mediated endocytosis requires Src-mediated tyrosine phosphorylation of cortactin [15], that regulates the interaction of cortactin with dynamin 2, a GTPase that has been implicated in the endocytic vesicles pinch-off [16]. A recent study demonstrated the essential role of Src-dependent tyrosine phosphorylation of cortactin and dynamin 2 in transferrin endocytosis [17]. All of these findings suggest that cortactin is an important component of the receptor-mediated endocytic machinery, regulating together with actin and dynamin the scission of clathrin pits from the plasma membrane.

The keratinocyte growth factor (KGF or FGF7) and the fibroblast growth factor 10 (FGF10), secreted by dermal fibroblasts, promote cell migration in keratinocytes [18]–[22]. Both these ligands act by binding to the keratinocyte growth factor receptor (KGFR), a splicing variant of FGFR2 expressed exclusively on epithelial cells [23]. Our previous studies about the intracellular trafficking and the endocytic pathway of KGFR upon KGF and FGF10 treatment, have demonstrated that both KGFR ligands induce KGFR internalization through clathrin-coated pits [24], [25]. Regarding the motogenic activity of KGF and FGF10, we have previously shown that KGFR ligands are able to induce a migratory, polarized phenotype in keratinocytes [22]: however, the FGF10-induced cell migration, as well as the translocation of focal adhesion components, such as paxillin, or of actin-binding proteins, such as cortactin, in ruffles and lamellipodia, was delayed compared to KGF [22]. This delayed motogenic effect of FGF10 correlated with a less intense, transient and delayed tyrosine phosporylation of cortactin [22].

The possible polarization of KGFR and of its endocytic trafficking at the leading edge upon ligand stimulation, as well as the possible involvement of cortactin and Src in the regulation of this process, have not been investigated yet. To address these points, we analyzed here the localization of KGFR during the motogenic response to the ligands and the role of receptor endocytosis in the process. We demonstrate Src-dependent and cortactin-dependent polarized localization of KGFR following treatment with the ligands. Moreover, we show that cortactin and KGFR colocalize at the plasma membrane protrusions and on early endosomes distributed at the leading edge of migrating cells and that this process is Src-dependent. Further, either inhibition of Src activity or siRNA depletion of cortactin or clathrin result in a block of KGFR internalization and, in turn, of receptor polarization. Finally, through a motility scratch assay we demonstrate that expression and polarization of the receptors are responsible for the increase of cell migration. Collectively, our results suggest that Src and cortactin play a crucial role in the KGFR endocytosis and subsequent receptor polarization at the leading edge of keratinocytes during migration promoted by KGF and FGF10.

## Results

### Src activity controls KGFR polarization at the leading edge of migrating cells

**Figure 1:**
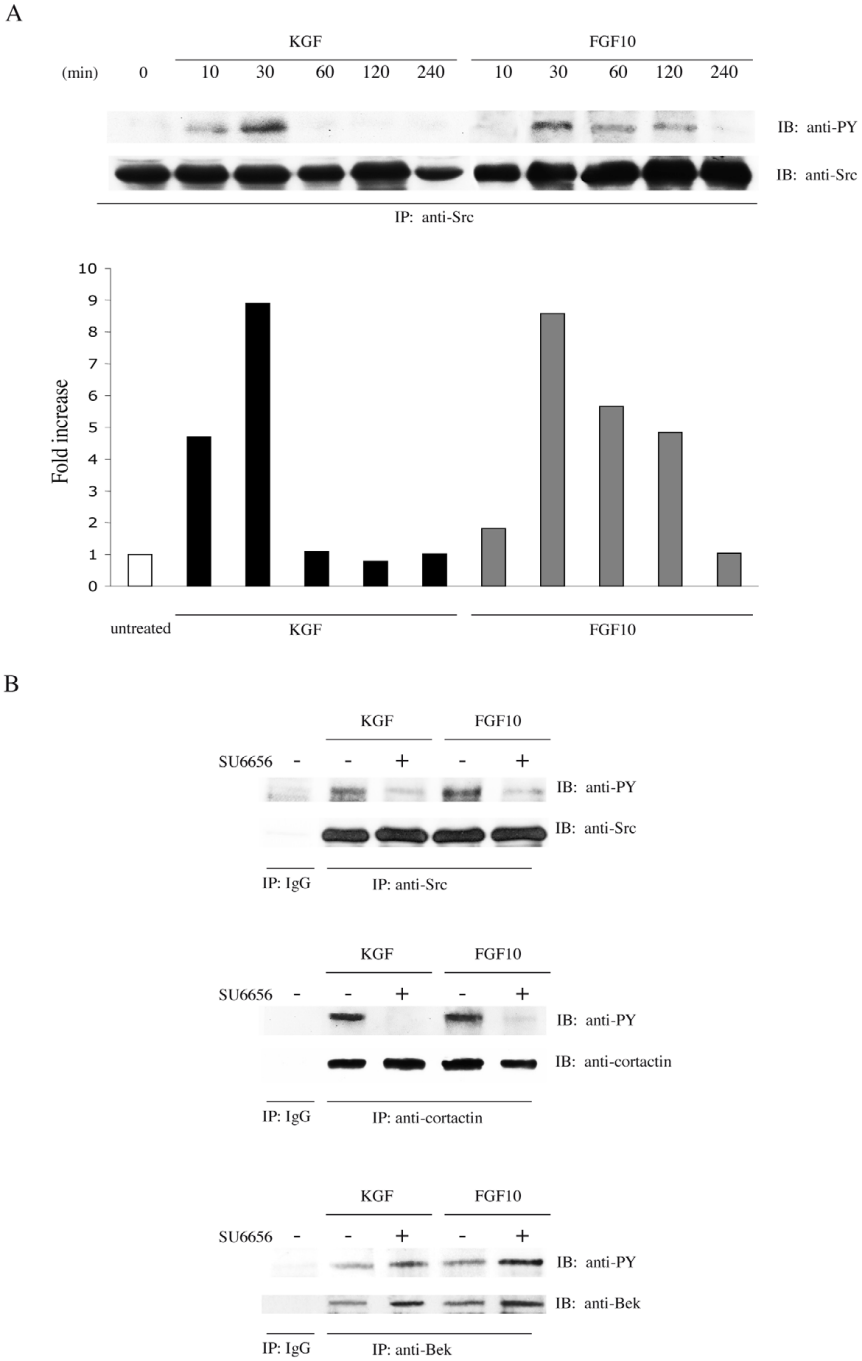
Src is tyrosine phosphorylated by KGFR ligand stimulation. A) HaCaT cells were serum starved and treated with KGF or FGF10 at 37°C for different time points. Immunoprecipitation with anti-Src antibodies and immunoblot with anti-phosphotyrosine antibody show that KGF treatment induces a rapid phosphorylation of Src, already evident after 10 minutes of stimulation and rapidly decreasing after 30 minutes. In FGF10-treated cells Src phosphorylation is detectable after 30 minutes of treatment, and gradually decreases, although still present up to 2 hours. The equal loading was assessed using anti-Src antibodies. The intensity of the bands was evaluated by densitometric analysis; the values from a representative experiment were normalized, expressed as fold increase with respect to the control value and reported as graph. B) HaCaT cells were pre-treated or not with the Src-specific inhibitor SU6656 and stimulated with KGF or FGF10 for 10 and 30 minutes, respectively. Immunoprecipitation with anti-Src, anti-cortactin or anti-Bek antibodies and immunoblot with anti-phosphotyrosine antibody show that the treatment with SU6656 blocks the tyrosine phosphorylation induced by KGFR ligands of Src and cortactin, but not of KGFR. The equal loading was performed using anti-src, anti-cortactin or anti-Bek antibodies.

Since cortactin is an important component of the receptor-mediated endocytosis machinery [12], [13], [15], and its function is dependent on tyrosine phosphorylation mediated by Src [8], to evaluate the role of cortactin and Src in KGFR internalization, we first determined whether Src would be tyrosine phosphorylated by KGFR activation. To this end, we used the human keratinocyte HaCaT cell line, spontaneously immortalized from a primary culture of keratinocytes [26], and we performed a biochemical analysis of Src phosphorylation in serum-starved cells treated with KGF or FGF10 at different time points, as reported in Materials and Methods. Immunoprecipitation with anti-Src antibodies and immunoblot with anti-phosphotyrosine antibody demonstrated that cells treated with KGF showed a rapid phosphorylation of Src, that was already evident after 10 minutes of stimulation and rapidly decreased after 30 minutes of treatment (Fig. 1A). In contrast, in FGF10-treated cells Src phosphorylation started after 30 minutes of treatment, then gradually decreased, but it was still present until 2 hours (Fig. 1A). The equal loading was assessed using anti-Src antibodies. Thus, KGF and FGF10 were both able to activate Src, although FGF10-induced Src phosphorylation resulted delayed with respect to that promoted by KGF. Since these kinetics of appearance of Src tyrosine phosphorylation corresponded to those we have previously observed for cortactin phosphorylation [22], here we decided to use 10 and 30 minutes of treatment with KGF and FGF10, respectively. To verify whether the tyrosine phosphorylation of Src was prevented by the specific Src-inhibitor SU6656, as expected [27], starved HaCaT cells were pre-treated or not with SU6656 and stimulated with KGF or FGF10, as above. Immunoprecipitation with anti-Src antibodies and immunoblot with anti-phosphotyrosine antibody revealed that SU6656 treatment inhibited tyrosine phosphorylation of Src (Fig. 1B). To confirm that Src activation was directly responsible for cortactin tyrosine phophorylation, immunoprecipitation with anti-cortactin antibodies and immunoblot with anti-phosphotyrosine antibody was performed on HaCaT cells, treated as above. As shown in Figure 1B, the SU6656 completely inhibited the tyrosine phosphorylation of cortactin induced by KGFR ligands, as expected [22]. To exclude that the block of cortactin tyrosine phosphorylation could be due to the inhibition of the kinase of the receptor, starved HaCaT cells, treated and stimulated as above, were immunoprecipitated with anti-Bek antibodies and immunoblotted with anti-phosphotyrosine antibody. Results showed in Figure 1B revealed that the treatment wih SU6656 did not inhibit the tyrosine phosphorylation of the receptor. Anti-Src, anti-cortactin and anti-Bek antibodies were used to assess equal loading. Taken together, these results show that, in our cellular model, the block of tyrosine phosphorylation of cortactin is due to a specific inhibition of Src.

**Figure 2:**
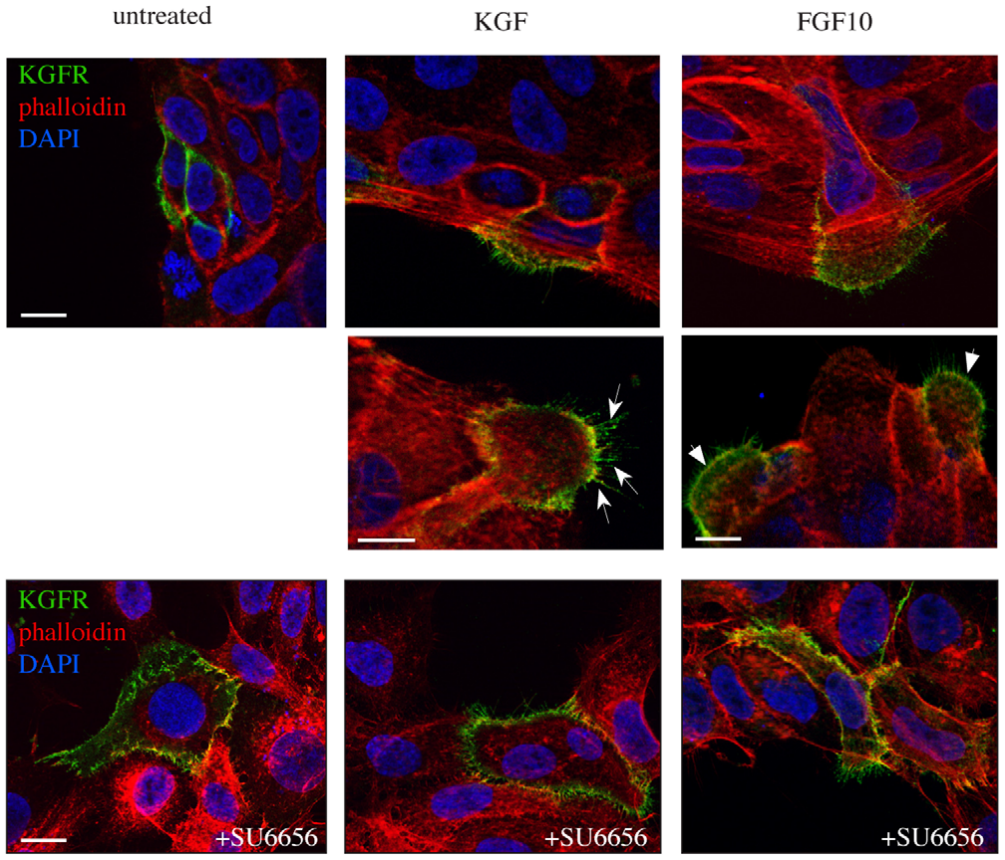
Src activity is responsible for KGFR polarization at the leading edge of migrating cells. HaCaT cells transiently transfected with the human KGFR (HaCaT KGFR) were treated with KGF or FGF10 and then immunostained at 4°C with an anti-Bek polyclonal antibodies, which recognize the extracellular portion of the receptor, and do not compete with the ligands for binding. Cells were then fixed, permeabilized, and stained with TRITC-conjugated phalloidin to visualize the actin cytoskeleton organization. Nuclei were stained with DAPI. Images are obtained, as described in Materials and Methods, by serial optical sectioning and 3D reconstruction of a selection of three out of the total number of sections: the selected sections are all central and crossing the nucleus visualized by DAPI. Results show that, in peripheral untreated HaCaT KGFR cells, the KGFR signal appears homogeneously distributed on the entire plasma membrane and the actin is not organized in typical migratory structures. After either KGF or FGF10 treatment, the KGFR signal appears polarized at the leading edge of migrating cells, where actin cytoskeleton is mainly organized in filopodia (arrows), but also in ruffles and small lamellipodia (arrowheads). In the presence of the Src inhibitor SU6656, the KGFR signal remains uniformly distributed on the plasma membrane, also upon ligand stimulation, and the actin is organized in few thin filopodia distributed over the entire cell surface. Bars: 10 µm.

Since we have previously demonstrated that both KGF and FGF10 induce migration of keratinocytes and translocation of cortactin to the leading edge of migrating cells [22], we wondered whether also the KGFR was polarized to the leading edge of migrating cells upon ligand stimulation. Unfortunately, the amount of KGFR in non transfected pre-confluent cells is not sufficient enough to be followed by conventional immunofluorescence methods, as previously reported by our group [25]; therefore, HaCaT keratinocytes were transiently transfected with the human KGFR (HaCaT KGFR) and treated with KGF or FGF10, as above. To selectively analyze the possible KGFR re-distribution on the cell surface after ligand stimulation, HaCaT KGFR cells were immunostained for 1 h at 4°C with an anti-Bek polyclonal antibodies, which recognize the extracellular portion of the two splicing variants KGFR/FGFR2b and FGFR2c and which do not compete with the ligands for binding to the receptor. To analyze the actin cytoskeleton remodeling, cells were then fixed and permeabilized, as described in Materials and Methods, and stained with TRITC-conjugated phalloidin, which specifically binds to filamentous actin. We focused our attention on cells overexpressing KGFR located at the periphery of the colonies, since we have previously observed that only these peripheral cells are able to assume the migratory phenotype in response to motogenic factors [22]. The results revealed that, in peripheral untreated HaCaT KGFR cells, the KGFR signal was distributed on the entire plasma membrane with no specific polarization, and the actin cytoskeleton was not organized in typical migratory structures (Fig. 2, upper panel). In contrast, after either KGF or FGF10 treatment, the KGFR signal appeared polarized at the leading edge of migrating cells (Fig. 2, upper and middle panels), where actin cytoskeleton was mainly organized in filopodia (Fig. 2, arrows in middle panel), but also in ruffles and small lamellipodia (Fig. 2, arrowheads in middle panel). Thus, KGFR is polarized to the leading edge of keratinocytes during both KGF- and FGF10-induced cell migration.

To analyze if the KGFR polarization would be dependent on Src activity, we performed parallel experiments in the presence of SU6656. The results showed that the Src inhibitor was able to block the receptor polarization induced by KGF and FGF10: in fact, even upon stimulation with the ligands, the KGFR signal remained uniformly distributed on the entire plasma membrane (Fig. 2, lower panels), as observed in unstimulated cells (Fig. 2, lower panel). Moreover, when Src kinase was inhibited, the actin cytoskeleton appeared organized mainly in thin filopodia evenly distributed over the entire cell surface (Fig. 2, lower panels). These results suggested a direct involvement of Src, probably through cortactin tyrosine phosporylation, in the induction of KGFR polarization during cell motility promoted by KGF and FGF10.

**Figure 3:**
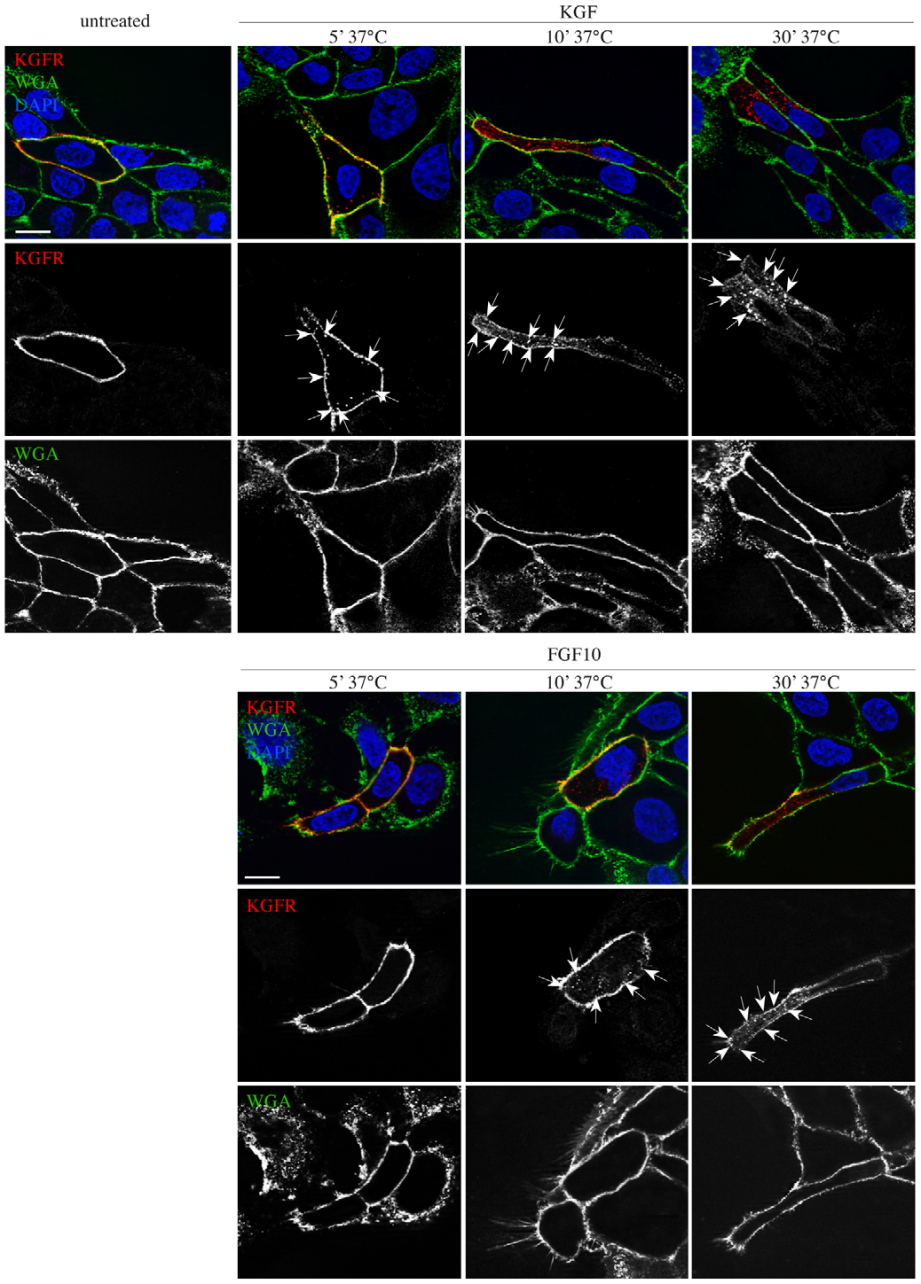
The KGFR polarization is dependent on receptor endocytosis. HaCaT KGFR cells were incubated at 4°C with the anti-Bek polyclonal antibodies to selectively stain the plasma membrane receptors and exclusively follow them during endocytosis, and then stimulated with KGF or with FGF10 for 5, 10 and 30 minutes at 37°C. The plasma membrane was decorated with the plasma membrane marker WGA-FITC. KGFR staining appears continuous and uniformly distributed on the cell surface of untreated cells, discontinuous on the plasma membrane and in some intracellular dots underlying the cell surface upon 5 minutes of KGF stimulation, and polarized at both the plasma membrane and in intracellular dots at the leading edge of migrating cells upon 10 and 30 minutes of KGF stimulation. After FGF10 stimulation the polarization appears delayed and evident only upon 30 minutes of treatment. The staining of the marker WGA appears uniformly distributed along the plasma membrane at all time points. Bars: 10 µm.

It has been reported that in Drosophila border cells motility requires RTKs to polarize at the leading edge, and that this spatially controlled localization is actively maintained by endocytosis followed by recycling of the receptors to the plasma membrane [28]. Therefore, to evaluate if also in our cell model the endocytosis and recycling could be responsible for KGFR polarization, we performed a time kinetic of the receptor relocalization at early time points upon KGF and FGF10 stimulation. To selectively stain the plasma membrane receptors and exclusively follow them during endocytosis, HaCaT KGFR cells were incubated at 4°C with the anti-Bek polyclonal antibodies, directed against the extracellular portion of KGFR, and then treated with KGF or FGF10 for 5, 10 and 30 minutes at 37°C, to induce receptor internalization. Moreover, to assess if the possible KGFR relocalization could be a real receptor polarization, KGFR staining at the plasma membrane was compared to that of the plasma membrane marker WGA-FITC. As shown in Figure 3, the KGFR staining was continuous and uniformly distributed along the entire cell surface in untreated cells. Upon 5 minutes of KGF stimulation the receptor staining appeared discontinuous on the plasma membrane and partially concentrated in intracellular dots underlying the entire cell surface (Fig. 3, arrows), suggesting receptor clustering in membrane pits and its internalization in early endocytic vesicles. Upon 10 and 30 minutes of stimulation with KGF, the receptor labeling appeared strongly polarized both at the plasma membrane and in intracellular dots, concentrated at the leading edge of migrating cells (Fig. 3, arrows). At each time point, the plasma membrane profile was assessed by the uniformly distributed staining of the marker WGA, showing that receptor polarization was a real effect (Fig. 3). Upon FGF10 stimulation, the KGFR polarization appeared slightly delayed and clearly visible only at 30 minutes of ligand treatment (Fig. 3, arrows). These results strongly suggest that the ligand-dependent polarization of KGFR is a direct consequence of its endocytosis and polarized recycling.

### KGFR and cortactin colocalize on the plasma membrane and in endosomes at the leading edge of migrating cells

**Figure 4:**
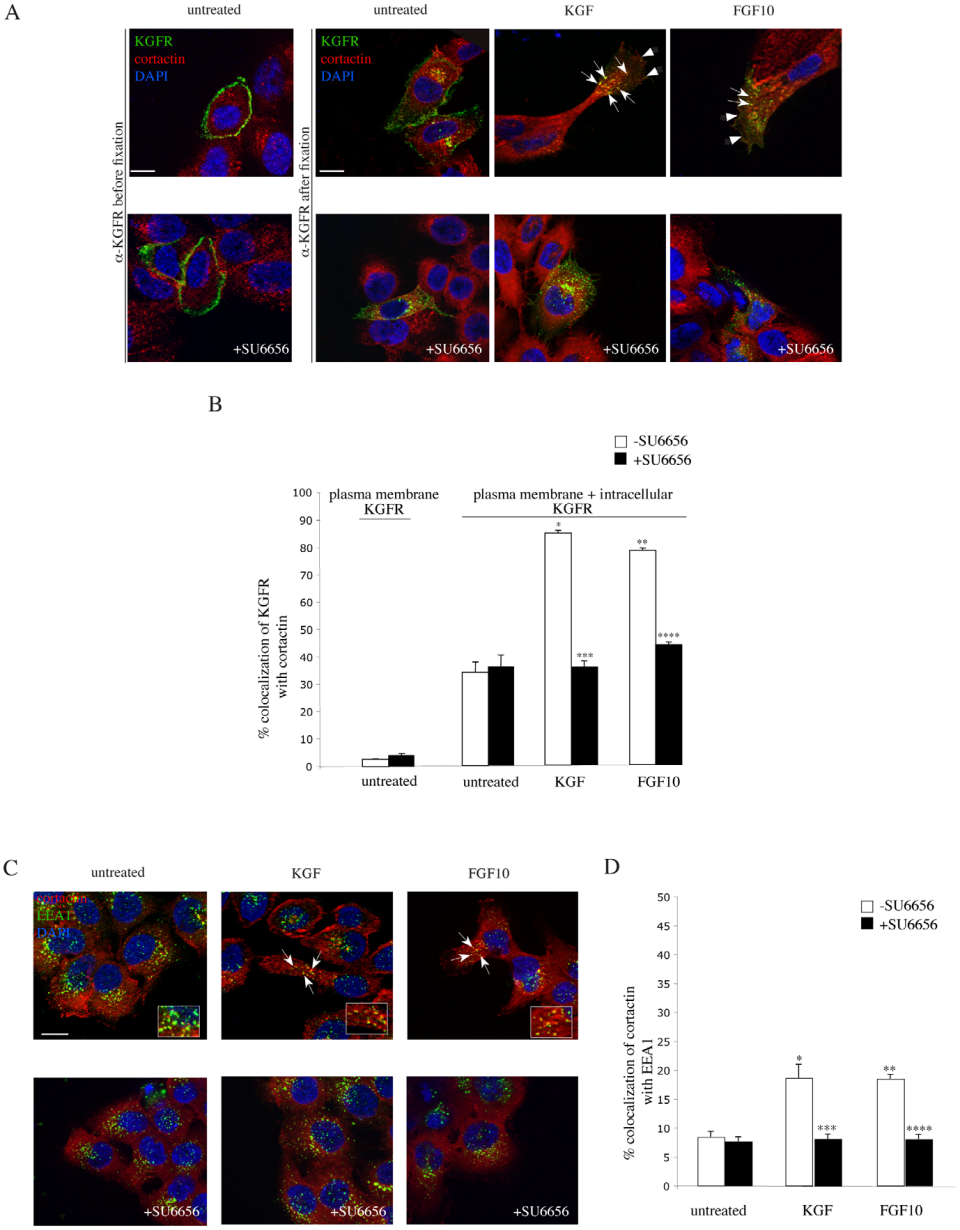
Src activity is required for KGFR and cortactin colocalization. A) HaCaT KGFR cells, treated or not with the growth factors, were incubated at 4°C with the anti-Bek polyclonal antibodies before cell fixation, as above, to selectively stain the plasma membrane KGFR, or with an anti-Bek monoclonal antibody after cell fixation and permeabilization, to visualize simultaneously the intracellular and plasma membrane KGFRs. Double immunofluorescence analysis, using anti-cortactin monoclonal antibody or anti-cortactin polyclonal antibodies, shows that in untreated cells the KGFR signal is localized along the entire surface of the cell plasma membrane, as well as in intracellular dots, and the cells do not show migratory features. The cortactin staining appears mainly localized on small intracellular dots dispersed throughout the cytoplasm, and only partially overlapping with KGFR positive dots. After treatment with either KGF or FGF10, cortactin and KGFR colocalization is significantly increased, evident not only in intracellular endocytic dots (arrows), but also at the level of the plasma membrane, where cortactin is translocated (arrowheads). Upon ligand treatment HaCaT KGFR cells located on the periphery of the colonies show a typical migratory phenotype, with a clearly defined leading edge, where the intracellular yellow dots stained for both cortactin and KGFR appear to be concentrated. Treatment with SU6656 reduces the colocalization between KGFR and cortactin, at a level comparable to that observed in untreated cells, and abolishes the migratory features. Images shown were obtained by 3D reconstruction of a selection of three out of the total number of the serial optical sections as reported in figure 2. Bars: 10 µm. B) Quantitative analysis of the percentage of colocalization of KFGR and cortactin was performed by serial optical sectioning and 3D reconstruction, as reported in Materials and Methods. Results are expressed as mean values +/- SE (standard errors): the percentage of colocalization was calculated analyzing a minimum of 50 cells for each treatment randomly taken from three independent experiments. Student's T test was performed and significance levels have been defined. *p<0,001 vs the corresponding untreated cells; **p<0,001 vs the corresponding untreated cells; ***p<0,001 vs the corresponding cells –SU6656; ****p<0,001 vs the corresponding cells –SU6656. C) Double immunofluorescence analysis, using anti-cortactin polyclonal antibodies and anti-early endosome antigene 1 (EEA1) monoclonal antibody, shows that the colocalization between cortactin and EEA1, already visible in untreated HaCaT cells, is increased upon KGF or FGF10 treatment and appears mostly relocalized in dots at the leading edge of migrating cells (arrows). The treatment with SU6656 significantly reduces the cortactin/EEA1 colocalization, as well as the migratory phenotype, upon either KGF or FGF10 stimulation. Images shown were obtained by 3D reconstruction of a selection of three out of the total number of the serial optical sections as reported in figure 2. Bars: 10 µm. D) Quantitative analysis of the percentage of colocalization of cortactin with EEA1 was performed by serial optical sectioning and 3D reconstruction as above. Results are expressed as mean values +/- SE; the percentage of colocalization was calculated analyzing a minimum of 50 cells for each treatment randomly taken from three independent experiments. Student's T test was performed and significance levels have been defined. *p<0,005 vs the corresponding untreated cells; **p<0,005 vs the corresponding untreated cells; ***p<0,001 vs the corresponding cells –SU6656; ****p<0,005 vs the corresponding cells –SU6656.

Since we have previously shown that cortactin translocates to the plasma membrane in correspondence to the surface areas towards the direction of the movement [22], and here we demonstrate that the KGFRs polarize to the leading edge of migrating cells (Fig. 2, 3), it was reasonable to suppose that cortactin and KGFR may colocalize. For this reason, we analyzed in detail the localization of KGFR and cortactin, and investigated whether and where the two proteins could colocalize during cell migration induced by KGF and FGF10. To this aim, HaCaT KGFR cells were treated or not with the ligands, as above, and the distribution of KGFR on the plasma membrane was visualized incubating cells with the anti-Bek polyclonal antibodies at 4°C before fixation, as above (Fig. 4A, upper panel, untreated). The intracellular and plasma membrane localization of KGFR was simultaneously assessed, after cell fixation and permeabilization, using an anti-Bek monoclonal antibody, which recognizes the intracellular portion of the receptor (Fig. 4A, upper panels). Cortactin localization was visualized using anti-cortactin monoclonal or polyclonal antibodies. Quantitative immunofluorescence analysis, performed as described in Materials and Methods, showed that in untreated cells the KGFR signal was localized along the entire surface of the cell plasma membrane (Fig. 4A, upper left panels), as well as on intracellular dots, probably corresponding to the receptor biosynthetic pathway and/or turnover pathway [29]. In these cells the cortactin staining appeared mainly localized on small intracellular dots dispersed throughout the cytoplasm (Fig. 4A, upper panels), as expected [9], and only partially overlapping with KGFR intracellular staining (Fig. 4B). Moreover, untreated cells did not show any typical migratory phenotype (Fig. 4A, upper panels), as expected. After treatment with either KGF or FGF10, for 10 and 30 minutes, respectively, the KGFR colocalization with cortactin was significantly increased (Fig. 4B) and visible not only in intracellular endocytic dots (Fig. 4A, upper panels, arrows), but also at the level of the plasma membrane, where the cortactin was translocated (Fig. 4A, upper panels, arrowheads). Furthemore, upon ligand treatment HaCaT KGFR cells located on the periphery of the colonies showed a typical migratory phenotype, with a clearly defined leading edge, where the intracellular yellow dots stained for both cortactin and KGFR appeared to be concentrated (Fig. 4A, upper panels). The treatment with SU6656 reduced the colocalization between KGFR and cortactin at a level comparable to that observed in untreated samples (Fig. 4B), and abolished the migratory features (Fig. 4A, lower panels), as expected [22]. Thus, Src activation is required for receptor/cortactin colocalization in polarized endocytic dots accumulated at the leading edge of migrating cells.

Since cortactin has been described to associate with endosomes [9]–[11] and here we demonstrate that KGFR polarization is the result of the receptor endocytosis and recycling (Fig. 3), we wondered if the KGFR and cortactin could colocalize in the early endosomal compartment when keratinocyte migration was triggered by KGFR ligand stimulation. To this aim, we first characterized the intracellular dots in which cortactin was clusterized when HaCaT KGFR cells were treated with KGF and FGF10. Quantitative immunofluorescence analysis, using anti-cortactin polyclonal antibodies and anti-early endosome antigene 1 (EEA1) monoclonal antibody, a specific marker of sorting endosomes, revealed that the percentage of colocalization of cortactin with EEA1 (8.4%) increased after treatment with both KGF (18.6%) and FGF10 (18.5%) (Fig. 4C, upper panels; 4D), and the double positive dots appeared mainly localized at the leading edge of migrating keratinocytes (Fig. 4C, upper panels, arrows). These results suggest that the intracellular dots distributed at the leading edge of migrating cells, in which cortactin and KGFR are clustered upon KGF- or FGF10-induced cell migration, as described above (Fig. 4A), correspond to the sorting endosomes. The treatment with SU6656 significantly reduced the cortactin/EEA1 colocalization (7.6%) upon either KGF (8%) or FGF10 (8%) stimulation, but did not interfere with the basal amount of cortactin localization in endosomes (Fig. 4C, lower panels; 4D). Thus, KGFR-mediated Src signaling, and consequent tyrosine phosphorylation of cortactin is able to induce an increased localization of cortactin in endosomes, where it colocalizes with KGFR.

**Figure 5:**
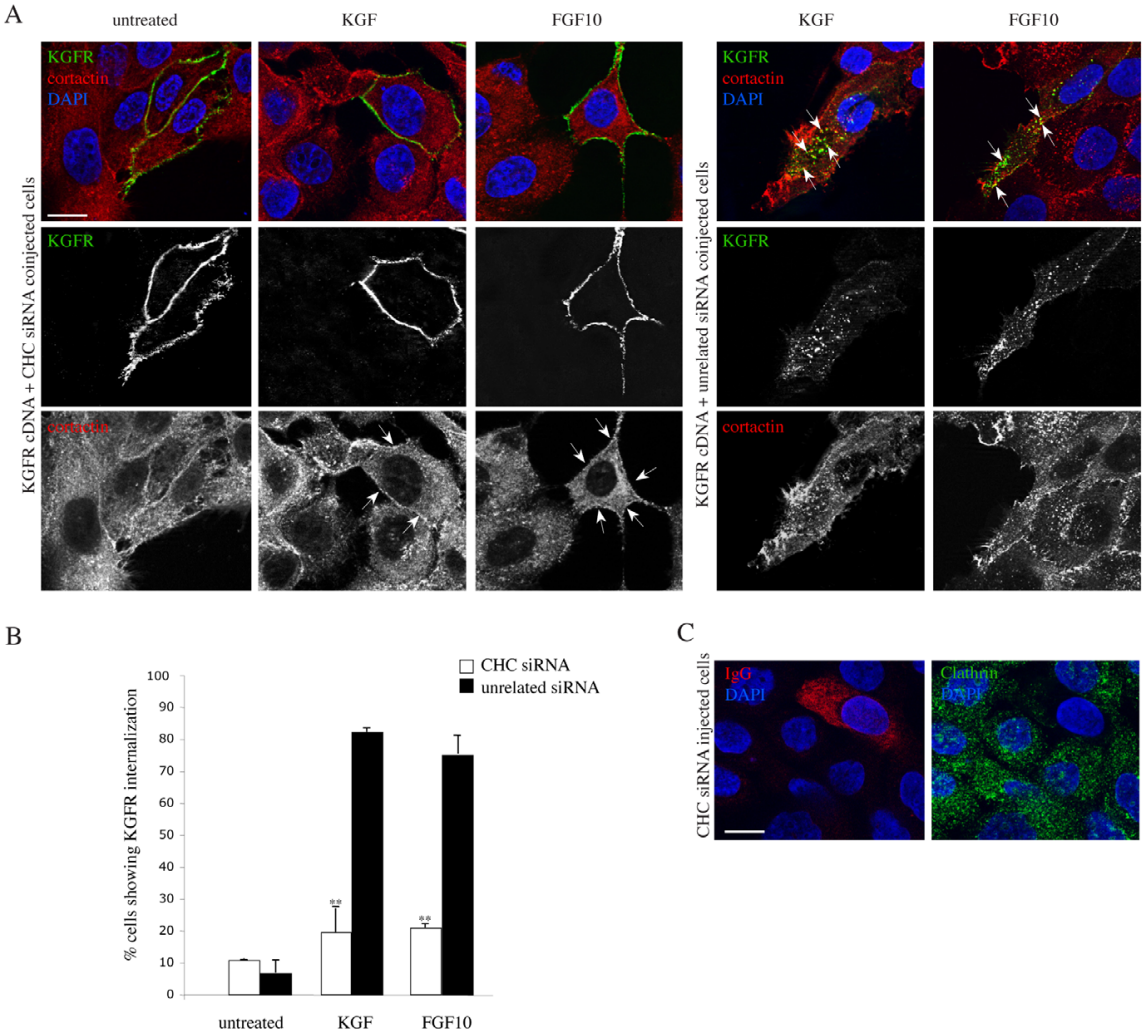
Clathrin depletion affects KGFR polarization and cortatin recruitment to the endosomes. A) HaCaT cells were coinjected with KGFR cDNA and CHC siRNA, to simultaneously obtain KGFR overexpression and CHC depletion, or with KGFR cDNA and an unrelated siRNA, as a control. After injection cells were serum starved, incubated at 4°C with the anti-Bek polyclonal antibodies, and treated with KGF or FGF10, as above. Upon KGF and FGF10 stimulation, in cells microinjected with CHC siRNA the receptor remains uniformly distributed on the plasma membrane and does not appear on intracellular dots, while the cortactin is dispersed throughout the cytoplasm and appears to be translocated just below the plasma membrane. In cells microinjected with the control siRNA, the KGFR is internalized and it colocalizes with cortactin in intracellular dots polarized at the leading edge of migrating cells. Bar: 10 µm. B) Quantitative analysis of percentage of HaCaT KGFR cells showing internalized KGFR was performed by counting 50 cells that overexpress KGFR for each condition, randomly taken from 10 microscopic fields in three different experiments, and values are expressed as the mean value ± standard errors (SE). C) HaCaT cells were coinjected with CHC siRNA and rabbit IgG to identify the microinjected cells. The immunofluorescence analysis was performed using anti-clathrin antibody: the punctate staining corresponding to clathrin-positive structures is reduced in microinjected cells compared to the surrounding uninjected cells. Bar: 10 µm.

To unequivocally demonstrate that the receptor polarization and consequent cortactin relocalization is a consequence of receptor endocytosis, we blocked the KGFR internalization by siRNA interference to selectively inhibit the clathrin-mediated pathway by silencing clathrin heavy chain (CHC). In fact, in our previous reports, we have demonstrated that, differently from other receptor tyrosine kinases, such as EGFR, the KGFR enter the cells only by a clathrin-dependent mechanism and that the CHC silencing is able to completely block its internalization [30], [31]. The efficiency of CHC depletion was assessed by coinjection of CHC siRNA and rabbit IgG to identify the microinjected cells. The immunofluorescence analysis with anti-clathrin antibody demonstrated that the punctate staining, corresponding to clathrin-positive pits and vesicles, was reduced in microinjected cells compared to the surrounding uninjected cells (Fig. 5C) or to cells injected with an unrelated siRNA (data not shown). To investigate the effect of CHC depletion on KGFR and cortactin polarization, HaCaT cells were coinjected with KGFR cDNA and CHC siRNA to simultaneously obtain KGFR overexpression and CHC depletion. Coinjection of KGFR cDNA and an unrelated siRNA was performed as a control. After injection cells were serum starved, incubated at 4°C with the anti-Bek polyclonal antibodies, and then treated with KGF or FGF10 as described above. Quantitative double immunofluorescence analysis showed that, upon ligand stimulation, in cells microinjected with CHC siRNA, the receptor remained uniformly distributed on the plasma membrane (Fig. 5A), and did not appear concentrated in intracellular dots, confirming that the receptor endocytosis is impaired. In these cells the cortactin appeared distributed throughout the cytoplasm and translocated below the plasma membrane (Fig. 5A, arrows). In contrast, in cells microinjected with the control siRNA, the KGFR was internalized and its colocalization with cortactin was visible in some intracellular dots polarized at the leading edge of migrating cells (Fig. 5A, arrows). Thus, following ligand stimulation, the intracellular KGFRs, which colocalize with cortactin, are those derived from the plasma membrane following internalization and the clathrin-mediated endocytosis is required for both receptor and cortactin polarization.

### Src signaling is required for KGFR endocytosis

**Figure 6:**
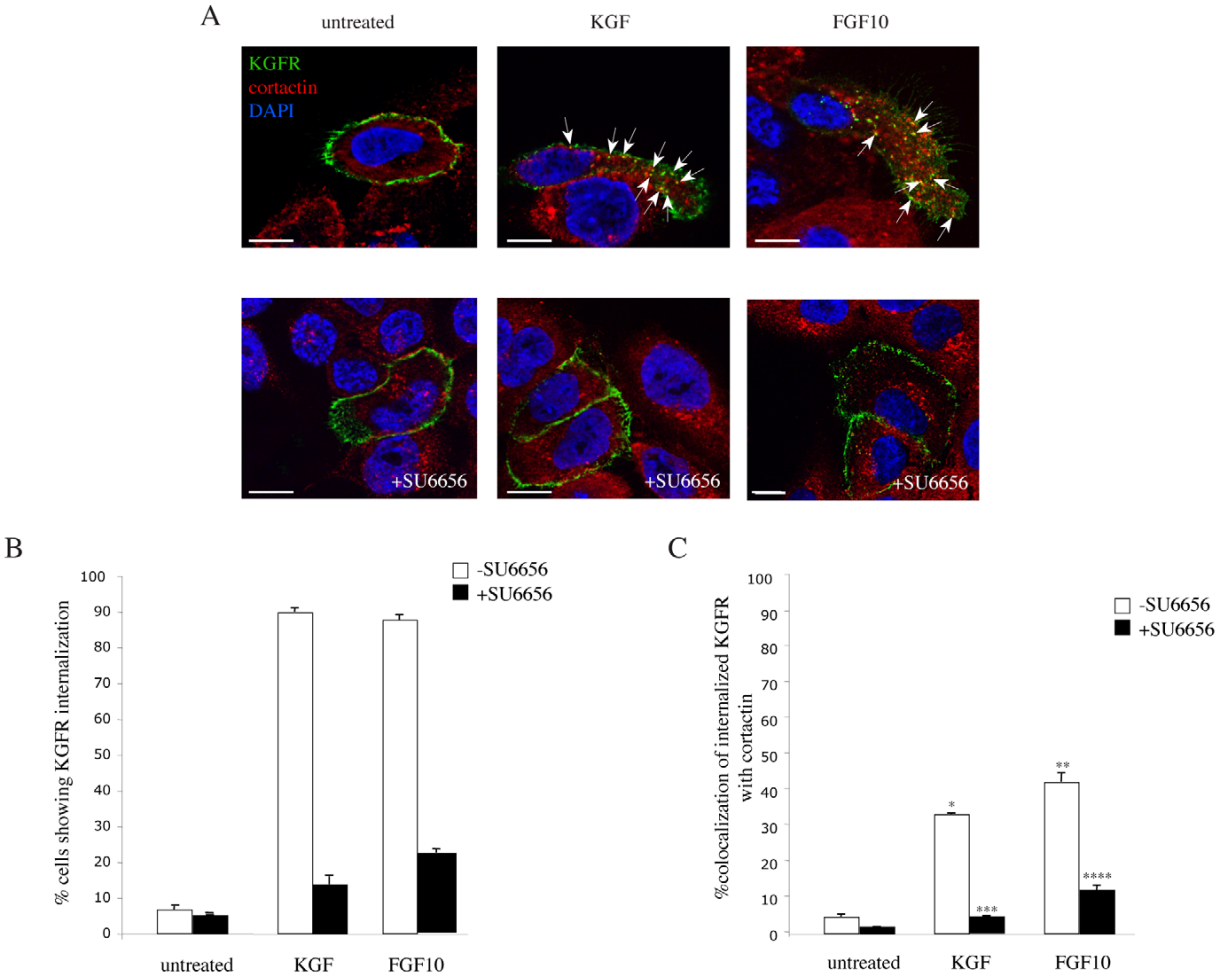
Src signaling is required for KGFR endocytosis. A) HaCaT KGFR cells were incubated at 4°C with the anti-Bek polyclonal antibodies, to selectively stain the plasma membrane receptors, and then treated with KGF or FGF10 to induce receptor internalization from the plasma membrane. Double immunofluorescence analysis, using anti-cortactin monoclonal antibody, shows that in untreated cells the KGFR signal appears uniformly distributed on the cell surface, while the cortactin signal is evident in dots dispersed throughout the cytoplasm, which correspond to sorting endosomes. Virtually no colocalization is observed between the two proteins. After KGF or FGF10 stimulation, HaCaT KGFR cells show a migratory phenotype and the internalized KGFR appear in endocytic dots polarized at the leading edge of migrating cells, in which the receptor significantly colocalizes with cortactin (arrows). Treatment with SU6656 is able to block the ligand-induced KGFR internalization, and consequently its colocalization with cortactin in endocytic dots: the receptor staining is uniformly distributed on the plasma membrane, while the cortactin labeling remains dispersed throughout the cytosol, as observed in untreated cells. Images shown were obtained by 3D reconstruction of a selection of three out of the total number of the serial optical sections, as reported in figure 2. Bars: 10 µm. B) Quantitative analysis of percentage of HaCaT KGFR cells showing internalized KGFR was performed by counting 100 cells that overexpress KGFR for each condition, randomly taken from 10 microscopic fields in three different experiments, and values are expressed as the mean value ± standard errors (SE). C) Quantitative analysis of the percentage of colocalization of KGFR with cortactin was performed as described above. The percentage of colocalization was calculated analyzing a minimum of 50 cells for each treatment randomly taken from three independent experiments. Results are expressed as mean values +/- SE. Student's T test was performed and significance levels have been defined. Student's T test was performed and significance level has been defined as above. *p<0,005 vs the corresponding untreated cells; **p<0,005 vs the corresponding untreated cells; ***p<0,001 vs the corresponding cells –SU6656; ****p<0,005 vs the corresponding cells –SU6656.

To evaluate if the ligand-dependent internalization of KGFR, and consequently its colocalization with cortactin in endosomes, requires Src-dependent signaling, HaCaT KGFR cells were incubated at 4°C with the anti-Bek polyclonal antibodies and then treated with KGF or FGF10, in the presence or not of SU6656, as above. Double immunofluorescence analysis, using anti-cortactin monoclonal antibody, showed that in untreated cells the KGFR signal appeared uniformly and exclusively distributed on the cell surface, while cortactin labeling was evident in dots dispersed throughout the cytoplasm (Fig. 6A, upper panel). Virtually no colocalization was observed between the two proteins (Fig. 6A, upper panel; 6C). After KGF or FGF10 treatment, HaCaT KGFR cells showed the typical migratory phenotype (Fig. 6A, upper panels), and the internalized KGFR appeared in dots polarized at the leading edge of migrating cells, in which the receptor significantly colocalized with cortactin (Fig. 6A, upper panels, arrows; 6C). In contrast, the presence of SU6656 was able to block the ligand-induced KGFR internalization (Fig. 6A, lower panels; 6B) and consequently its colocalization with cortactin in intracellular dots (Fig. 6A, lower panels; 6C). In fact, upon ligand treatment the receptor staining remained uniformly distributed on the plasma membrane, while the dotted cortactin labeling appeared no longer polarized, but rather dispersed througouth the cytosol, as observed in untreated cells (Fig. 6A, lower panel). Moreover, in the presence of SU6656, HaCaT KGFR cells did not show the typical, ligand-induced migratory phenotype (Fig. 6A, lower panels). Thus, upon KGF and FGF10 stimulation, the KGFR internalization and intracellular colocalization with cortactin are Src signaling-dependent events.

### Cortactin depletion inhibits KGFR internalization and polarization

**Figure 7:**
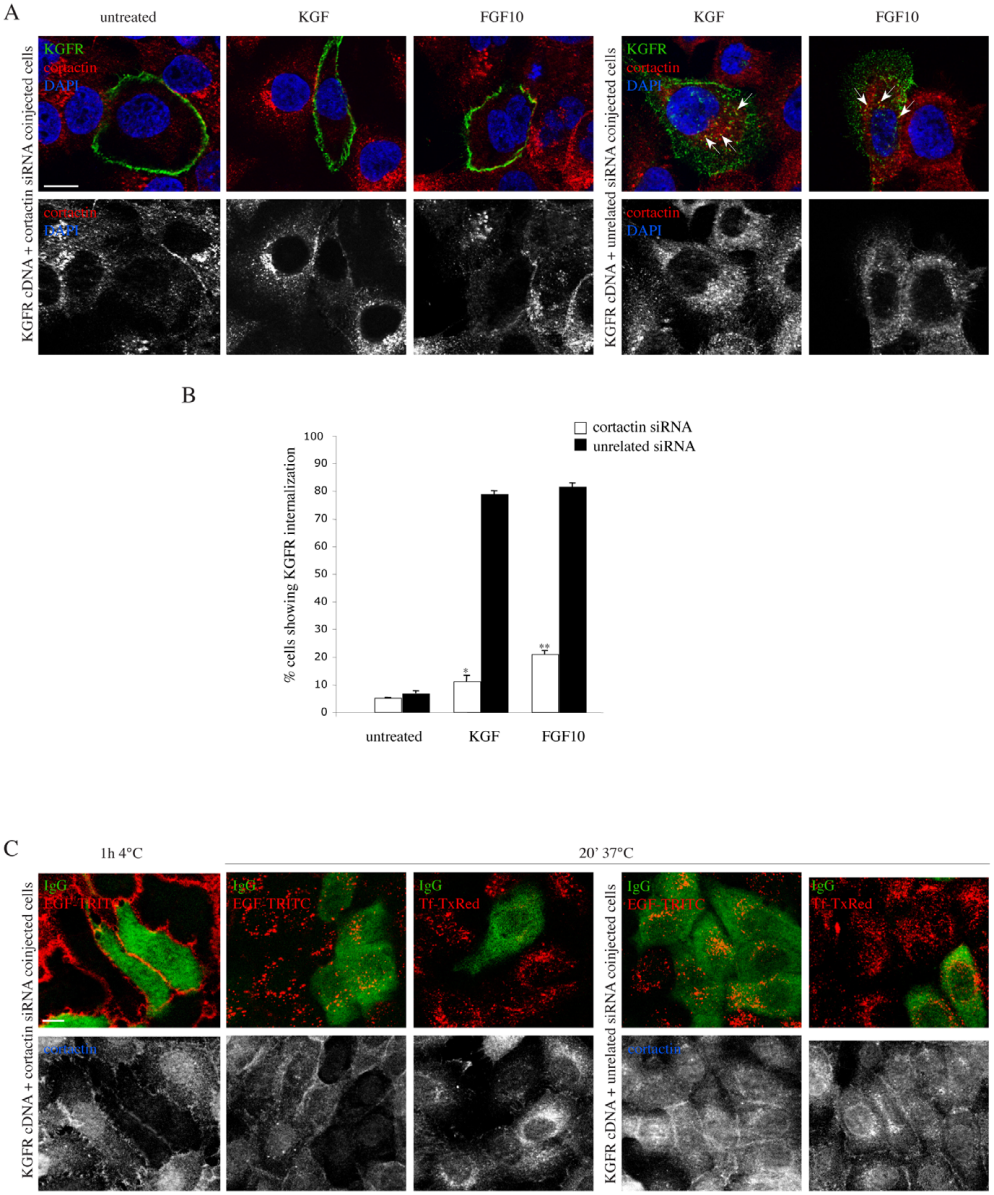
Cortactin depletion inhibits KGFR internalization and polarization. A) HaCaT cells were coinjected with cortactin siRNA and KGFR cDNA, to simultaneously induce cortactin silencing and KGFR overexpression. Control cells were injected with an unrelated siRNA. After injection cells were incubated at 4°C with anti-Bek polyclonal antibodies and treated with KGF or FGF10, as described above. Quantitative double immunofluorescence analysis, using anti-cortactin monoclonal antibody, shows that in cortactin siRNA/KGFR cDNA coinjected cells, KGFR signal is evident, while the cortactin signal appears strongly decreased if compared to the surrounding uninjected cells in the same microscopic fields or to control cells injected with unrelated siRNA (compare upper panels to lower panels). Upon ligands treatment, in cortactin-depleted cells KGFR signal remains uniformly distributed on the plasma membrane, while in cells microinjected with unrelated control siRNA, expressing cortactin, the KGFR appears internalized and its colocalization with cortactin is evident, as well as their polarization at the leading edge of migrating cells (arrows). Images shown were obtained by 3D reconstruction performed as reported in figure 2. Bar: 10 µm. B) Quantitative analysis of the percentage of KGFR internalization was performed as above. Results are expressed as mean values +/- SE and Student's T test was performed and significance level has been defined as above. *p<0,005 vs the corresponding untreated cells; **p<0,0001 vs the corresponding untreated cells. C) HaCaT cells were coinjected with a mixture of cortactin siRNA and rabbit IgG, to identify the microinjected cells, and then treated with EGF-TRITC or Transferrin-Texas Red (Tf-TxRed) for 1 h at 4°C or for 20 minutes at 37°C before fixation and permeabilization. Triple immunofluorescence analysis, using anti-cortactin monoclonal antibody, shows that in cells microinjected with the cortactin siRNA very low levels of cortactin staining were detectable, Tf internalization was strongly impaired, while EGF uptake appeared unaffected, if compared to uninjected cells or to cells injected with unrelated siRNA. Bar: 10 µm.

To demonstrate the possible, direct functional role of cortactin in regulating the KGFR endocytosis and its consequent polarization to the leading edge of migrating cells, we analyzed the effect of cortactin depletion on the ligand-induced KGFR internalization. To this aim, HaCaT cells were coinjected with a mixture of cortactin siRNA and KGFR cDNA, to simultaneously induce cortactin silencing and KGFR overexpression. Microinjection with an unrelated siRNA was performed as control. After injection, cells were incubated at 4°C with the anti-Bek polyclonal antibodies, and treated with KGF or FGF10, as described above. Quantitative double immunofluorescence analysis showed that, in cells overexpressing KGFR, cortactin depletion was highlighted by a strong decrease in signal intensity of the specific, dotted staining for cortactin, if compared to the surrounding uninjected cells in the same microscopic field or to control cells injected with unrelated siRNA (compare upper panels to lower panels in Fig.7A). Upon KGF or FGF10 treatment, in cortactin-depleted cells KGFR signal remained uniformly distributed on the plasma membrane (Fig. 7A, upper panels). In contrast, in cells microinjected with unrelated control siRNA, expressing cortactin, the KGFR appeared internalized and its colocalization with cortactin was evident, as well as their polarization at the leading edge of migrating cells (Fig. 7A, upper panels, arrows; 7B).

Since the specific requirement of cortactin in the regulation of clathrin-mediated endocytosis is still debated, and it has been reported that cortactin depletion does not affect clathrin-dependent endocytosis of EGFR [32], we analyzed in our cell system the effect of cortactin depletion on the internalization of EGFR and of the specific clathrin-dependent endocytosis marker Tf. To this end, HaCaT cells were coinjected with a mixture of cortactin siRNA and rabbit IgG, to identify the microinjected cells, and then treated with EGF-TRITC or Transferrin-Texas Red (Tf-TxRed) for 20 minutes at 37°C, to induce their internalization. Triple immunofluorescence showed that, in cells microinjected with the cortactin siRNA, in which very low levels of cortactin staining were detectable (Fig. 7C, lower panels), Tf internalization was strongly impaired, while EGF uptake appeared unaffected, if compared to uninjected cells or to cells injected with unrelated siRNA (Fig. 7C, upper panels). Thus, in our cellular model, cortactin silencing is able to inhibit Tf uptake and ligand-dependent endocytosis of KGFR, but not that of EGFR. These results strongly suggest a direct cargo-specific functional role of cortactin in the control of clathrin-dependent internalization and receptor polarization during cell migration.

### Polarized expression of KGFR enhances ligand-dependent cell migration

**Figure 8:**
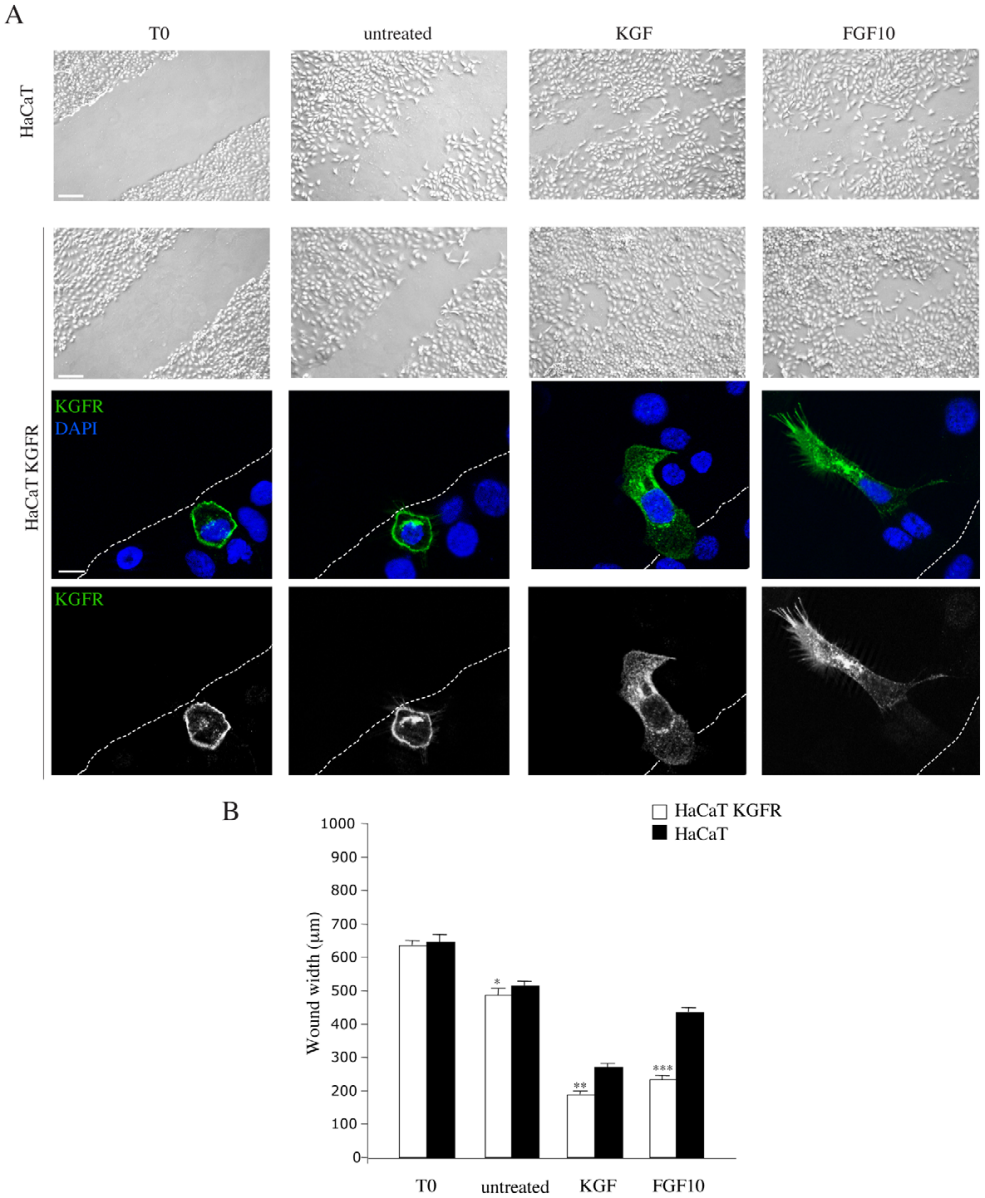
KGFR expression and polarization are involved in cell motility. A) HaCaT KGFR and HaCaT cells were seeded on coverslip and grown until confluence. A cell-free area was introduced in a monolayer of cells using a steril tip and then cells were immediately fixed (T0) or allowed to migrate from the edge of the scratch for 20 h at 37°C in the presence or not of KGF or FGF10. The cell-free area, evident in samples at time 0 (T0) from the scratch, is only partially repopulated in untreated cells. KGF stimulation induces a more intense cell migration if compared to FGF10 in HaCaT cells; KGFR overexpression induces a significant increase of cell migration upon both growth factors stimulation. Bar: 80 µm. Immunofluorescence analysis using anti-Bek antibodies in HaCaT KGFR cells shows some cells that clearly overexpresses KGFR. The receptor staining is uniformly distributed on the plasma membrane in unstimulated cells or in cells fixed at time 0 (T0) from the scratch, but is polarized at the leading edge of migrating cells that repopulate the scratch area upon KGF of FGF10 stimulation. Bar: 10 µm. B) Cells migration was quantified measuring the mean gap distance between the edges of the scratch area as reported in Materials and Methods. Student’s T test was performed and significance level has been defined as above. * NS vs the corresponding untransfected cells; **p< 0,0001 vs the corresponding untransfected cells; ***p<0,0001 vs the corresponding untransfected cells.

To evaluate if the KGFR expression and its polarization would be directly responsible for KGF- or FGF10-induced cell motility, we analyzed the effect of KGFR overexpression on HaCaT cell migration using the “scratch assay”. Briefly, a cell-free area was introduced in a monolayer of HaCaT KGFR and HaCaT cells, as previously described [22], and then cells were allowed to migrate from the edge of the scratch for 20 hours at 37°C in the presence of KGF or FGF10. Cell migration was quantified measuring the mean gap distance between the edges of the scratch area. As shown in Figure 8, HaCaT cells migrated faster in the presence of KGF than FGF10, as previously reported [22], and the overexpression of KGFR induced a significant enhancement of the migratory behaviour upon both ligands stimulation (Fig. 8A,B). In addition, immunofluorescence analysis, using anti-Bek antibodies, indicated that in those cells showing clearly KGFR overexpression, the receptor staining appeared polarized at the leading edge of migrating cells that invade the scratch area upon KGF or FGF10 stimulation (Fig. 8A, lower panels), while it was uniformly distributed on the cell plasma membrane, either in the absence of growth factor stimulation and in cells fixed at time 0 (T0) (Fig. 8A, lower panels). Thus, KGFR expression and even more its polarization are crucial for KGF and FGF10 induced cell migration.

## Discussion

The cortical actin cytoskeleton is a dynamic structure that regulates many cellular processes, including cell migration and endocytosis, by interaction with components of the plasma membrane [33]. Recently, a large number of observations have demonstrated that endocytosis is responsible for the polarized localization of RTKs to the leading edge of migrating cells and that receptor recycling is crucial for maintaining signaling localized, suggesting a complex link of function between these two processes [3]–[5]. Among a variety of candidates, the Src substrate actin-binding protein cortactin would seem the most suitable to play the role of a link between endocytosis and migration. In fact, besides its well known effect in regulating the actin cytoskeleton dynamic during cell migration [8], it appears to play a crucial role in the control of clathrin-dependent endocytosis [12], [13], [15]. The present study started from previous reports from our group showing that cortactin is involved in KGF- and FGF10-induced cell migration [22], and that the KGFR internalization triggered by the two ligands occurs by clathrin coated-pits [24], [25]. Here, we provided several lines of evidence indicating that Src activity and cortactin are required for the KGFR endocytosis and its polarization to the leading edge of migrating human keratinocytes upon KGF and FGF10 stimulation.

The direct evidence of the involvement of cortactin in KFGR internalization comes out from our observations that the downregulation of the protein, obtained by using microinjection of cortactin siRNA, specifically impairs the endocytosis of KGFR, as well as that of the specific clathrin-dependent endocytosis marker Tf. These results are in agreement with previous findings indicating that microinjection of anti-cortactin antibodies [12] and cortactin siRNA [13] inhibited transferrin and LDL uptake, demonstrating a role for cortactin in clathrin-mediated endocytosis, that is the exclusive internalization route followed by KGFR [30]. In contrast, also in our cell system, the cortactin depletion does not affect the EGF uptake, consistent with previous observations [32] suggesting that cortactin is not required for EGFR endocytosis. These contrasting results related to the two different receptor tyrosine kinases, KGFR and EGFR, might strongly suggest a cargo-specific functional role of cortactin in the control of clathrin-dependent internalization.

Interestingly, we found here that cortactin partially localizes on endocytic dots labeled with EEA1, as previously described [9], and that this localization increases upon KGF or FGF10 stimulation, similarly to the results previously reported describing the association of cortactin with endosomal structures following treatment with transferrin [11]. In agreement with a published report showing the cortactin/CXC chemokine receptor colocalization in endosomes during this receptor endocytic trafficking [10], we observed that cortactin colocalizes with internalized KGFRs in endosomes and that also this colocalization increases after KGF or FGF10 stimulation, suggesting that this actin-binding protein could finely regulate the early stages of ligand-dependent endocytosis of KGFR. Since the involvement of actin in membrane traffic is not restricted to the plasma membrane, but also affects the motility of endosomes [8], we hypothesize that, also in our cell model, a correct actin coat on endosomes, regulated by cortactin at the leading edge of migrating keratinocytes, is important for the correct intracellular transport of the receptor from and to the plasma membrane.

Cortactin is a substrate of Src and Src is involved in the trafficking of RTKs [34]-[36] and, in particular, of FGFRs [37]: in fact, Src regulates the activation, the signaling and the intracellular transport of FGFR1 [38], [39]. Here we analyzed if and how Src activation could be involved in the regulation of KGFR internalization and polarization during cell migration. Our biochemical results on the Src tyrosine phosphorylation revealed that both KGF and FGF10 are able to activate the Src kinase, although FGF10 effect was delayed with respect to that promoted by KGF. A possible explanation for the kinetics differences, already observed in our previous analysis of the cortactin phosphorylation [22], may be related to the different physiological heparin requirement for the binding of the two factors to the same receptor, as we have previously proposed [22], suggesting that the concentration and composition of HSPGs of the extracellular matrix coud regulate differently the affinity binding of KGF and FGF10 to KGFR, modulating the biological response to these growth factors.

Interestingly, recent findings have indicate that differences in the kinetics of Src activation correlated to alternative endocytic trafficking of FGFR1 upon FGF2 and NCAM ligand stimulation [39]. Therefore, it is reasonable to suppose that the differences in Src phosphorylation found upon KGF and FGF10 stimulation would be responsible for the previously described alternative endocytic fate of KGFR induced by its two ligands [30]. However, in this present study, we demonstrate that Src activity regulates also the internalization of KGFR and consequent receptor polarization during cell migration induced by both KGF and FGF10. In fact, the Src inhibitor SU6656 was able to interfere with the polarization of activated KGFR at the leading edge of migrating cells blocking the clathrin-dependent internalization of the receptor. These results are in contrast to those obtained by Sandilands et al. [38] about FGFR1, in which Src inhibition blocks the receptor in endocytic vesicles preventing its presentation on the cell surface, but are in agreement with the findings obtained by Broudy et al. [34] and, more recently, by Marcotte et al. [40], which demonstrated that the treatment with the Src inhibitors PP1 and PP2 blocks the SCF-induced clathrin-dependent internalization of c-Kit and significantly inhibits that of the EGFR, respectively. Because we and others have proposed that the internalization mechanisms of FGFR1 and FGFR2b/KGFR may be quite different in terms of clathrin involvement [24], [30], [41], it is possible that the role of Src in the uptake of receptors of the FGFR family may be highly variable. Our results, pointing to the crucial function of the internalization process in determining polarized localization of KGFR and its signaling at the leading edge of migrating cells, as unequivocally shown through inhibition of its clathrin-mediated endocytosis, are in accordance with previous reports showing that, during migration of Drosophila border cells, continuous internalization events regulate the polarity of EGFR and PVR and that this receptor polarization is essential for the motogenic response [28], [42], [43]. Consistently with these results, we demonstrate here, through a functional motility assay, that receptor expression and polarization increase the migratory behaviour.

Following endocytosis and targeting to early endosomes, receptors (integrins as well as RTKs) are recycled back to the plasma membrane via two distinct recycling pathways, both responsible for receptor polarized relocalization. The short-loop pathway is a Rab4GTPase-dependent rapid recycling from early endosomes, while the long-loop pathway is controlled by Rab11GTPase and implies the receptor transit through the perinuclear recycling compartment [5]. Because we have previously demonstrated that, once internalized by the ligands, KGFR follows two alternative endocytic routes, i.e. KGF targets the receptor to the degradative pathway, whereas FGF10 induces KGFR sorting to the recycling compartment [30], future work will be focused to characterize the recycling pathways responsible for KGFR polarization during cell motility. In particular, based on the present observations that KGF and FGF10 have different ability to induce Src tyrosine phosphorylation, it would be interesting to verify if the two KGFR ligands might be able to mediate the receptor polarization through receptor targeting to different endocytic recycling pathways in migratory cells.

## Materials and Methods

### Materials

Human recombinant KGF, anti-phosphotyrosine monoclonal antibody (clone 4G10) and anti-cortactin monoclonal antibody (clone 4F11) were purchased from Upstate Biotechnology (Lake Placid, NY, USA). Recombinant FGF10 was from PeproTech (London, UK). Src inhibitor SU6656 was obtained by Calbiochem (San Diego, CA, USA).). Goat anti-mouse Alexa Fluor 350, recombinant EGF-TRITC and Tranferrin-Texas Red were from Molecular Probes (Eugene, OR, USA). Anti-Bek (C-8) and anti-clathrin monoclonal antibodies, Anti-Bek (C-8) monoclonal antibody anti-cortactin, anti-Src (SRC 2), anti-Bek (H80) and anti-Bek (C-17) polyclonal antibodies were from Santa Cruz Biotechnology (Santa Cruz, CA, USA). Heparin, TRITC-phalloidin, FITC-conjugated lectin wheat germ agglutinin (WGA) and DAPI were from Sigma (Sigma Chemicals, St. Louis, MO). Anti-EEA1 monoclonal antibody was obtained from Biosciences (San Josè, CA, USA). FITC-conjugated goat anti-rabbit IgG and goat anti-mouse IgG were obtained from Cappel Research Products (Durham, NC, USA), Texas Red-conjugated goat anti-mouse IgG and goat anti-rabbit IgG were from Jackson Immunoresearch Laboratories (West Grove, PA, USA).

### Cells and treatments

The human keratinocyte cell line HaCaT, spontaneously immortalized from a primary culture of keratinocytes [26], were cultured in Dulbecco's Modified Eagle's Medium (DMEM; Euroclone, Pero, MI, Italy), supplemented with 10% fetal bovine serum (FBS) and antibiotics. Cells were transiently transfected with pCI-neo vector containing human KGFR WT using jetPEI TM DNA Trasfection Reagent (Polyplus-trasfection, New York, NY, USA) according to manufacturer's instructions. To induce Src and cortactin tyrosine phosphorylation, KGFR internalization and cell migration, HaCaT cells were serum starved for 4 h and then treated with 50 ng/ml KGF or with 50 ng/ml FGF10 + 0,3 µg/ml heparin and then warmed to 37°C for different times. To exclusively label KGFRs on the plasma membrane, HaCaT KGFR cells were incubated for 1 h at 4°C with the anti-Bek polyclonal antibodies (H-80, 1∶50 in DMEM medium), directed against the extracellular portion of KGFR, which do not compete with the ligands for binding to the receptor. To selectively follow the internalized receptors during KGFR endocytosis, HaCaT KGFR cells were previously starved for 12 h at 37°C, incubated for 1 h at 4°C with anti-Bek polyclonal antibodies H-80 and then treated with 50 ng/ml KGF for 10 min or with 50 ng/ml FGF10 + 0,3 µg/ml heparin for 30 min at 37°C. To induce EGF internalization, cells were serum starved and then stimulated for 1 h at 4°C or for 20 minutes at 37°C with 50 ng/ml EGF-TRITC. To induce transferrin internalization, cells were serum starved for 4 h and then treated with 50 µg/ml Transferrin-Texas Red (Tf-TxRed) for 20 minutes at 37°C. To visualize the cell surface, plasma membranes were decorated with FITC-WGA at 4°C before fixation and permeabilization.

For inhibition of Src-family-specific protein tyrosine kinases cells were preincubated with SU6656 (5 µM) for 1 h before stimulation with KGF or FGF10 in the presence of the same inhibitor at 37°C in prewarmed medium.

### Scratch assay

HaCaT KGFR and HaCaT cells were seeded at 1,5×10^5^ cells on coverslips and grown until confluence. Confluent cells were serum starved for 12 h and then a standardized cell-free area was introduced by scraping the monolayer with a sterile tip, as previously described [22]. Some coverslips were fixed and photographed immediately after scratching, representing a T0 control. After intensive wash, cells were incubated for 20 h in the presence of KGF or FGF10. Cells were fixed with 4% paraformaldehyde and processed for immunofluorescence. Both phase contrast and immunofluorescence images were taken using an ApoTome System (Zeiss, Oberkochen, Germany) connected with an Axiovert 200 inverted microscope (Zeiss). Cell migration was quantitated by measuring the gap distance between the scratch edges (three different measures for each photograph), using the Axiovision software (Zeiss). The results are expressed as the mean values of three independent experiments ± SE. p values were calculated using Student's t test and significance level has been defined as p<0.05.

### Immunoprecipitation and Western blot analysis

Subconfluent cultures of HaCaT cells, treated with KGF or FGF10 and with SU6656, were lysed in a buffer containing 50 mM Tris–HCl pH 7.4, 150 mM NaCl, 1% NP-40, 1 mM EDTA, supplemented with protease inhibitors (10 µg/ml aprotinin, 10 µg/ml leupeptin, 2 mM PMSF), and phosphatase inhibitors (1 mM sodium orthovanadate, 20 mM sodium pyrophosphate, 50 mM sodium fluoride). One milligram of total protein was immunoprecipitated with 4 µg/ml of anti-Src (SRC 2), anti-cortactin or anti-Bek polyclonal antibodies. Immunocomplexes, aggregated with 50 µl of γ-bind protein-G sepharose (Amersham Biosciences, Uppsala, Sweden), were washed four times with 0.5 ml of buffer. The pellets were boiled in Laemmli buffer for 5 min, and the protein resolved under reducing conditions by 8% SDS–PAGE and transferred to nitrocellulose (PROTRAN, Schleider and Schuell, Keene, NH, USA). The membranes were blocked with 5% nonfat dry milk in PBS 0.1% Tween 20 and incubated with anti-phosphotyrosine monoclonal antibody diluted 1∶1000 for 1 h at 25°C, followed by goat anti-mouse-HRP secondary antibody and enhanced chemiluminescence detection (ECL; Amersham Biosciences, Arlington Heights, IL, USA). To estimate the protein equal loading, the membranes were rehydrated by being washed in PBS–Tween 20, stripped with 100 mM mercaptoethanol and 2% SDS for 30 min at 55°C and reprobed with anti-Src, anti-cortactin or anti-Bek polyclonal antibodies, diluted 1∶1000. Densitometric analysis was performed using Photoshop Quantity One Program (Bio-Rad Laboratories GmbH, Munich, Germany). Briefly, the signal intensity for each band was calculated and the background subtracted from experimental values. The resulting values were then normalized, expressed as fold increase with respect to the control value and visualized as graph.

### Immunofluorescence

HaCaT cells, grown on coverslips, were fixed with 4% paraformaldehyde in PBS for 30 min at 25°C, followed by treatment with 0.1 M glycine in PBS for 20 min at 25°C and with 0.1% Triton X-100 in PBS for additional 5 min at 25°C to allow permeabilization. Cells were then incubated for 1 h at 25°C with the following primary antibodies: anti-Bek (H-80, 1∶50 in PBS), anti-cortactin polyclonal antibodies (1∶50 in PBS) and anti-Bek (C-8, 1∶50 in PBS), anti cortactin (4F11, 1∶50 in PBS), anti-EEA1 (1∶50 in PBS) monoclonal antibodies. The primary antibodies were visualized, after appropriate washing with PBS, using goat anti-rabbit IgG-FITC (1∶400 in PBS), goat anti-mouse IgG-FITC (1∶50 in PBS), goat anti-mouse IgG-Texas Red (1∶200 in PBS) and goat anti-rabbit IgG-Texas Red (1∶200 in PBS) and goat-anti mouse Alexa fluor 350 (1∶50 in PBS) for 30 min at 25°C. Actin cytoskeleton was visualized using TRITC-phalloidin (1∶100 in PBS). Nuclei were stained with 4′,6-diamidino-2-phenylindol (DAPI) (1∶10.000 in PBS). Coverslips were finally mounted with mowiol for observation.

To assess the extent of colocalization of fluorescence signals, cells were scanned in a series of 0.5 µm sequential sections with an ApoTome System (Zeiss) connected with an Axiovert 200 inverted microscope (Zeiss); image analysis was then performed by the Axiovision software (Zeiss). Images obtained by 3D reconstruction of a selection of three out of the total number of the serial optical sections are shown in each figure: the selected sections are all central and crossing the nucleus visualized by DAPI staining. Quantitative analysis of the extent of colocalization was performed using Zeiss KS300 3.0 Image Processing system (Zeiss). The mean +/- standard error (SE) percent of colocalization was calculated analyzing a minimum of 50 cells for each treatment randomly taken from three independent experiments. p values were calculated using Student's t test and significance level has been defined as p<0.05.

Quantitative analysis of percentage of HaCaT KGFR cells showing receptor internalization was performed by counting 100 cells for each condition, randomly taken from 10 microscopic fields in three different experiments, and values are expressed as the mean value ± standard errors (SE).

### siRNA-mediated downregulation of cortactin and CHC

The cortactin-specific short interference RNA (siRNA), which specifically knocks down cortactin gene expression, and control siRNA, which does not lead to the specific degradation of any cellular message, were purchased from Santa Cruz Biotechnology. Starved HaCaT cells were microinjected with an Eppendorf microinjector (Eppendorf, Hamburg, Germany) and an inverted microscope (Zeiss, Oberkochen, Germany). Injection pressure was set at 80–100 hPa and the injection time at 0.5 s. A mixture of 100 mM RNA interfering for cortactin and 100 ng/µl cDNA coding for KGFR or RNA interfering for cortactin and 3 mg/ml rabbit IgG have been microinjected into the cells to simultaneously induce RNA interference, and consequent cortactin silencing and KGFR overexpression. Cells were then starved for 12 h at 37°C, incubated for 1 h at 4°C with anti-Bek polyclonal antibodies H-80, treated with KGF for 10 min or with FGF10 for 30 min at 37°C as above and finally processed for immunofluorescence microscopy. For clathrin heavy chain (CHC) depletion and block of clathrin mediated endocytosis, a mixture of 100 nM CHC si RNA (Santa Cruz) and 100 ng/µl KGFR cDNA was microinjected into HaCaT cells to simultaneously induce CHC silencing and KGFR overexpression. Cells were then starved and treated with KGF or FGF10, as above, and processed for immunofluorescence. The efficiency of CHC depletion was assessed by parallel injection of a mixture of 100 nM CHC si RNA and 3 mg/ml rabbit IgG.
